# Molecular epidemiology and clinical characteristics of carbapenem-resistant *Klebsiella pneumoniae* bloodstream and pneumonia isolates

**DOI:** 10.1128/spectrum.00631-25

**Published:** 2025-07-09

**Authors:** Phillip B. Cox, Jocelyn Qi-Min Teo, Derrick E. Fouts, Thomas H. Clarke, Felicia Ruffin, Vance G. Fowler, Joshua T. Thaden, Andrea Lay-Hoon Kwa

**Affiliations:** 1 Division of Infectious Diseases, Duke University School of Medicine 12277 , Durham, North Carolina, USA; 2 Division of Pharmacy, Singapore General Hospital 37581, Singapore, Singapore; 3 SingHealth Duke-NUS Pathology Academic Clinical Programme, Singapore, Singapore; 4 J Craig Venter Institute 6946, Rockville, Maryland, USA; 5 Singhealth Duke-NUS Medicine Academic Clinical Programme, Singapore, Singapore; 6 Emerging Infectious Diseases Program, Duke-National University of Singapore Medical School, Singapore, Singapore; Universita degli Studi dell'Insubria, Varese, Italy

**Keywords:** carbapenem, *Klebsiella pneumoniae*, antibiotic resistance

## Abstract

Appropriate antibiotic therapy in patients with carbapenem-resistant *Klebsiella pneumoniae* (CRKp) infections often requires knowledge of resistance mechanisms. Therefore, we aimed to define the clonal diversity, mechanisms of carbapenem resistance, potential for carbapenem resistance transmissibility, virulence gene repertoire, and potential clinical impact of these genetic elements in patients with CRKp bloodstream infection and/or pneumonia, which are associated with high mortality. Clinical data and CRKp isolates at a site in Singapore and in the United States were examined. Whole-genome sequencing and bioinformatic analysis of CRKp isolates were performed. In total, 125 patients (*n* = 118 from Singapore; *n* = 7 from the United States) were included. Clonal diversity was high as 58 sequence types were identified. Carbapenemases were present in 109 (87%) of the CRKp isolates; KPC-, OXA-, and NDM-type enzymes were identified in significant quantity. The majority (87%) of carbapenemases were encoded on conjugative or mobilizable plasmids and thus potentially transmissible to other strains. Of the 16 CRKp isolates without a carbapenemase, the predominant predicted mechanism of carbapenem resistance was extended-spectrum β-lactamases along with truncation of porins *ompK35* and/or *ompK36*. Patients with carbapenemase-producing CRKp strains had increased in-hospital mortality. Recurrent CRKp infections were primarily due to either the same CRKp genomic group or similar carbapenemase-encoding conjugative plasmids within a different *K. pneumoniae* strain. Our findings suggest that in CRKp isolates causing serious infections, carbapenem resistance arises through diverse mechanisms, is easily transmissible between isolates, and impacts patient outcomes. These findings highlight the importance of diagnostic tools to identify mechanisms of carbapenem resistance in the clinical setting.

**IMPORTANCE:**

To design more effective therapies and better understand treatment failure in patients with carbapenem-resistant *Klebsiella pneumoniae* (CRKp) infections, we must identify the mechanisms of carbapenem resistance and their impact on patient outcomes. However, prior studies are often limited by the inclusion of CRKp that are colonizers or from non-severe infections, a focus on a single general mechanism (i.e., presence of carbapenemases), lack of whole-genome sequencing to fully characterize the underlying genetic architecture of CRKp strains, and no analyses of associations between resistance mechanisms and clinical outcomes. Here, we attempt to address some of these gaps by sequencing a large set of invasive CRKp isolates to characterize the underlying clonal diversity, mechanisms of carbapenem resistance, potential carbapenemase transmissibility, virulence gene repertoire, and the possible impact of these genetic elements on patient mortality.

## INTRODUCTION

Infections with carbapenem-resistant *Klebsiella pneumoniae* (CRKp) are an urgent health threat. *K. pneumoniae* is a common pathogen that causes approximately 10% of nosocomial infections (1), and antibiotic resistance drives particularly poor patient outcomes. Mortality rates in patients with CRKp bloodstream infections (BSIs) as high as 50%–60% have been reported, which is four- to eightfold higher than that in carbapenem-susceptible strains of *K. pneumoniae* (2, 3). In addition to the increased mortality, resistance to carbapenem antibiotics has been shown to drive up both healthcare costs and hospital length of stay (4, 5). Due to the poor clinical and economic outcomes associated with CRKp infections, as well as the dearth of antibacterials to treat them, the World Health Organization listed carbapenem-resistant *Enterobacteriaceae* like CRKp as Priority 1: Critical pathogens where additional research and drug development are needed (6).

Treating CRKp infections is challenging due to both antibiotic resistance and the diverse mechanisms of resistance that are employed (7–11). To design more effective therapies and better understand the mechanisms of treatment failure, it is necessary to understand the mechanisms of carbapenem resistance in patients with CRKp infections and how such mechanisms impact patient outcomes. With this in mind, we aimed to define the mechanisms of carbapenem resistance and potential mechanisms of carbapenem resistance transmissibility in a diverse set of clinical CRKp isolates that are primarily from patients in Singapore. The genomic epidemiology of CRKp in Singapore has been previously described in detail in several publications (12, 13). These prior studies included a large proportion of non-infection-causing colonizing isolates, and CRKp isolates driving invasive infections have been less well understood. Therefore, we focused on isolates causing bloodstream infection or pneumonia, as these syndromes are common and associated with particularly high mortality (14–17). In addition to the mechanisms of carbapenem resistance, the clinical impact of important genomic elements in invasive CRKp isolates—such as the underlying clonal background, capsule type, and virulence gene content—has not yet been explored in this patient population. An additional aim of this study was to explore the impact of these CRKp genetic elements on patient mortality.

## MATERIALS AND METHODS

### Study population

Patients were enrolled at Singapore General Hospital in Singapore (both CRKp bloodstream infections and pneumonia) and at Duke University Hospital in Durham, North Carolina, United States (CRKp bloodstream infections only). Carbapenem resistance was defined as resistance to at least one carbapenem antibiotic (meropenem, imipenem, or ertapenem) using the Clinical and Laboratory Standards Institute breakpoints. In Singapore, bacterial isolates were initially collected at the institution’s Microbiology Laboratory and kept at the Pharmacy Research Laboratory’s carbapenem non-susceptible gram-negative isolates repository as part of an informal surveillance study. Bloodstream and pneumonia isolates were identified from this repository consisting of isolates collected since 2006. The study was exempt from SingHealth Institutional Review Board review (2020/2583), as it involved only the analyses of an anonymous existing data set that did not comprise individually identifiable information. At Duke, the patient clinical data and bacterial isolates were obtained from the Duke Blood Stream Infection Biorepository. The Blood Stream Infection Biorepository contains prospectively collected clinical data and blood culture isolates from >4,500 unique inpatients at Duke University Hospital with monomicrobial gram-negative bacterial BSI since 2002. Consent was obtained from all study participants, and the study was approved by the Duke University Institutional Review Board. For the primary analyses, only one CRKp isolate was considered from each patient.

### Whole-genome sequencing and bioinformatics analyses

Clinical isolates from frozen stocks were streaked onto Luria-Bertani (LB) solid media plates and grown at 37°C overnight. Cultures for genomic DNA isolation were prepared by inoculating LB broth with a single colony and growing overnight at 37°C with orbital shaking at 225 revolutions per minute. DNA was isolated from overnight cultures with the Easy DNA Kit (Invitrogen, K180001). Libraries were prepared for sequencing with Illumina Nextera XT kits and sequenced on an Illumina NextSeq 500 with paired 150-base sequence reads. Approximately 75 libraries were loaded into two lanes and pooled with 1% PhiX spiked in. The average genome coverage was 192×. Each read set was assembled individually with *SPAdes* (18) and annotated with the National Center for Biotechnology Information Prokaryotic Genome Annotation Pipeline (19). A phylogenetic tree was generated using MASH average nucleotide identity (ANI) distances (20) with 100,000 16mers used to build the sketch file and Gaussian Genome Representative Selector with Prioritization (21) using average-linkage clustering to make the tree. Multilocus sequence typing (MLST), capsule type, *ompK35/36* mutations, the number of antibiotic resistance genes and classes, virulence genes, and virulence gene score were determined with Kleborate (22). Recurrent CRKp isolates were considered the same genomic group if the two strains had the same sequence type (ST) and ANI greater than 99%. ANI was calculated with MASH (20). The genomic context of the carbapenemase (i.e., chromosomal vs plasmid), as well as the potential transmissibility of plasmid-encoded enzymes (i.e., conjugative, mobilizable, and non-mobilizable), was determined with MOB-suite (23). A conjugative plasmid contains the complete set of genes and DNA features (i.e., the origin of transfer, a DNA relaxase, a type IV coupling protein, and the type IV secretion system) needed for plasmid transfer to another strain. A mobilizable plasmid is missing the mate-pair formation marker but can be transferred with the help of a conjugative plasmid that is present in the same cell. A non-mobilizable plasmid cannot be transferred via conjugation. MOB-suite was also used to determine the similarity of plasmids found within recurrent CRKp strains. While long-read sequencing was not performed, MOB-suite attempts to reconstruct the specific plasmid content from genome assemblies. A pair of CRKp isolate plasmids (i.e., plasmids within the initial and recurrent CRKp isolates from the same patient) was determined to be similar if the plasmid contigs were mapped to the same reference assembly and contained the same carbapenemase enzyme.

### Determination of carbapenemase production

Whole-genome sequencing was used to identify CRKp isolates with a carbapenemase gene. If such a gene was detected, then this was assumed to be the mechanism of carbapenem resistance. If no such gene was detected, then a modified Hodge test was performed to identify carbapenemase activity. The modified Hodge test was performed as in a published protocol (24).

### Quantitative reverse transcription PCR

Gene expression of *ompK35* and *ompK*36 was quantified for carbapenemase-negative isolates using quantitative reverse transcription PCR (qRT-PCR). Total cellular RNA was extracted from exponential-phase LB (Miller) broth cultures (1st Base, Singapore) using the Qiagen RNeasy Mini Kit (Qiagen, Germany) with DNase I pre-treatment according to the manufacturer’s recommendations. Total RNA concentrations and purity were determined using NanoDrop One Microvolume UV-Vis Spectrophotometer (Thermo Scientific, USA). Reverse transcription reactions were conducted using the Bio-Rad iScript cDNA synthesis (Bio-Rad, USA) kit with an amount of RNA standardized to 1 µg per reaction. Gene expression was determined using the Bio-Rad Universal iTaq Universal SYBR Green Supermix kit (Bio-Rad, USA) with the CFX384 Real-Time PCR Detection System (Bio-Rad, USA) with primers detailed in Table S1. Amplifications were performed in triplicates from two separate RNA preparations. Quantification of target genes was normalized to the level of the housekeeping *rpoB* gene, an endogenous reference commonly used for normalizing transcription levels (25). Relative gene expression was calculated as the fold change in expression of the analyzed strains compared to *K. pneumoniae* ATCC 13883 control strain (a strain known to express both proteins) using the 2^−ΔΔCT^ method (ΔΔ*C_T_* = [*C_T_ − C_TrpoB_*]_studied strain_ − [*C_T_ − C_TrpoB_*]_control strain_, where CT is the cycle threshold] (26). Additional qRT-PCR details are provided in the Supplemental Methods.

### Statistical analyses

Continuous variables were reported as means with SDs or medians with interquartile ranges (IQR) and compared with *t*-tests or median tests, respectively. Dichotomous variables were analyzed as counts and percentages, using Fisher’s exact or *χ*^2^ tests where appropriate. Univariable and multivariable logistic regression models were generated to determine the factors associated with total mortality (i.e., death prior to hospital discharge from any cause), attributable mortality (i.e., death due to CRKp infection prior to hospital discharge), and the presence of septic shock. Variables in the multivariable regression models included patient age, sex, source of positive culture (respiratory tract vs bloodstream), geography (Singapore vs United States), presence of a carbapenemase enzyme, and bacterial virulence score. All data analyses were conducted using RStudio version 4.3.1 (Boston, MA). *P*-values less than 0.05 were considered significant.

## RESULTS

### Patient cohort

In total, 125 patients with CRKp were included in this study, with 77 (62%) from the bloodstream and 48 (38%) from the lower respiratory tract (Table 1). Patients with CRKp bloodstream infections, relative to CRKp lower respiratory infections, differed with respect to age (bloodstream: mean 61 years [SD 17]; lower respiratory tract: 67 years [15]; *P* = 0.03) and sex (bloodstream: female 34/77 [44%]; lower respiratory tract: female 10/48 [21%]; *P* = 0.01). Most of the patients were enrolled in Singapore (118/125 [94%]). There were no statistically significant differences in complications or outcomes between the two groups.

**TABLE 1:** Characteristics of patients and bacteria in the study cohort^*a*^

Characteristics	Blood *n* (%) *N* = 77	Lower respiratory *n* (%) *N* = 48	*P*-value
Age in years (mean [SD])	61 (17)	67 (15)	**0.03**
Sex			**0.01**
Female	34 (44)	10 (21)	
Male	43 (56)	38 (79)	
Site			**0.04**
Singapore	70 (91)	48 (100)	
United States	7 (9)	0 (0)	
Infection year			0.54
2010	1 (1)	0 (0)	
2012	4 (5)	0 (0)	
2013	10 (13)	10 (21)	
2014	10 (13)	5 (10)	
2015	15 (19)	6 (13)	
2016	9 (12)	7 (15)	
2017	7 (9)	8 (17)	
2018	11 (14)	6 (13)	
2019	10 (13)	6 (13)	
Patient outcomes			
Total mortality	30 (39)	27 (56)	0.07
Attributable mortality	20 (26)	16 (33)	0.42
Septic shock	16 (21)	18 (38)	0.06

^*a*^ *P*-values less than 0.05 are indicated in bold.

### CRKp sequence types

**Fig 1:**
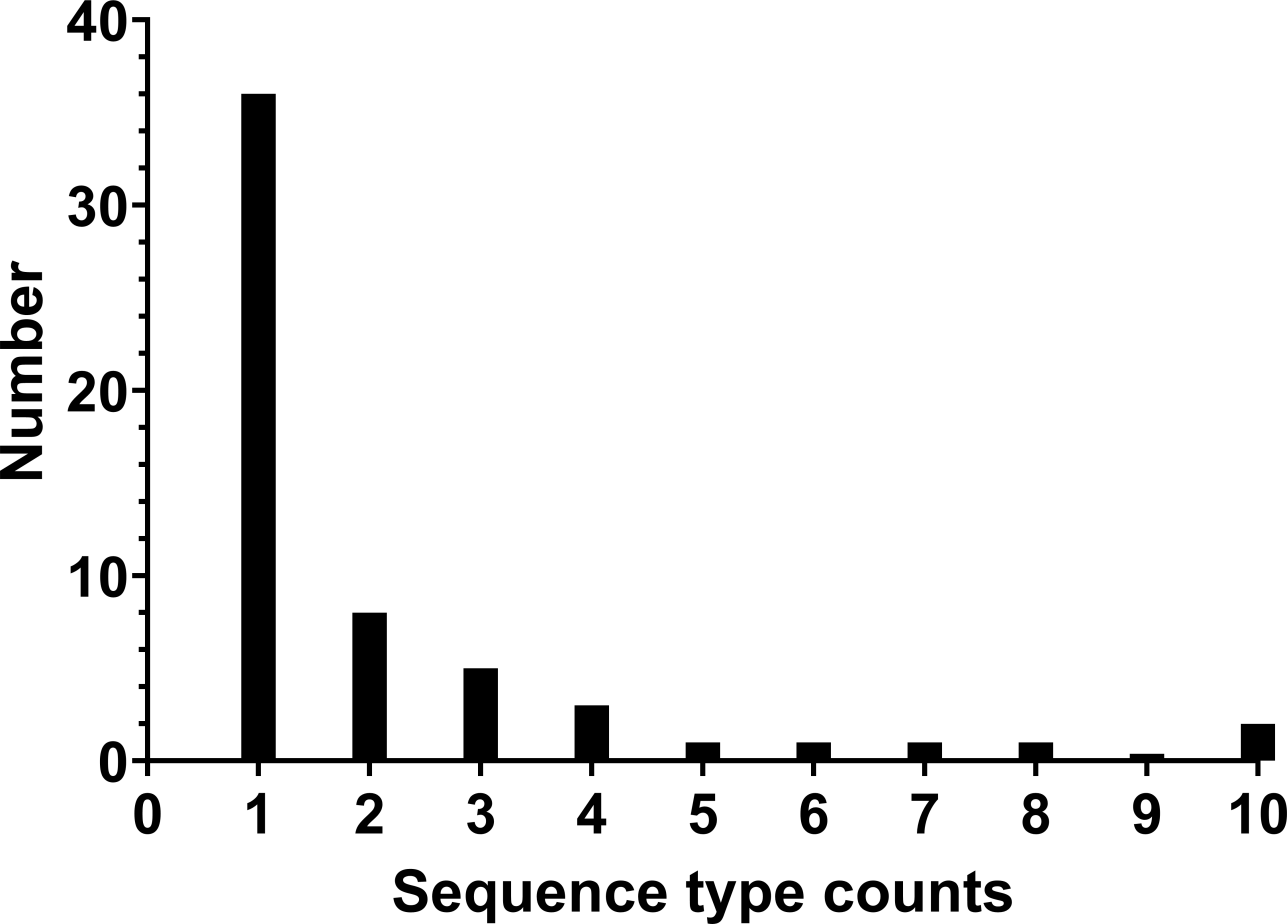
ST distribution of CRKp in this study. The x-axis is the number of CRKp isolates within each ST (i.e., ST count 1 corresponds to STs with only one CRKp isolate in this study), while the y-axis is the number of such occurrences. The majority of STs were unique and only identified through one CRKp isolate in this study.

MLST was performed on the CRKp isolates. Significant genetic variation within the CRKp cohort was identified. Of the 58 STs identified within the study population, 37 (64%) contained just a single CRKp isolate (Fig. 1). Only six sequence types occurred more than five times: ST11, ST14, ST147, ST15, ST16, and ST231 (Table 2). ST147 was only found in patients with BSI (*P* = 0.02). The remaining five STs were distributed between patients with CRKp bloodstream and lower respiratory tract infections.

**TABLE 2:** ST distribution of CRKp in this study^*a*^

ST	Blood (*n*)	Lower respiratory (*n*)	*P*-value
ST11	5	2	0.71
ST14	4	6	0.18
ST147	8	0	**0.02**
ST15	3	2	1.00
ST16	5	5	0.51
ST231	4	2	1.00

^*a*^ MLST of the CRKp isolates was performed, and STs with >5 occurrences are represented in this table. ST147 was only found in patients with CRKp BSI (*P* = 0.02). *P*-values less than 0.05 are indicated in bold.

### Carbapenemase-producing CRK

**Fig 2:**
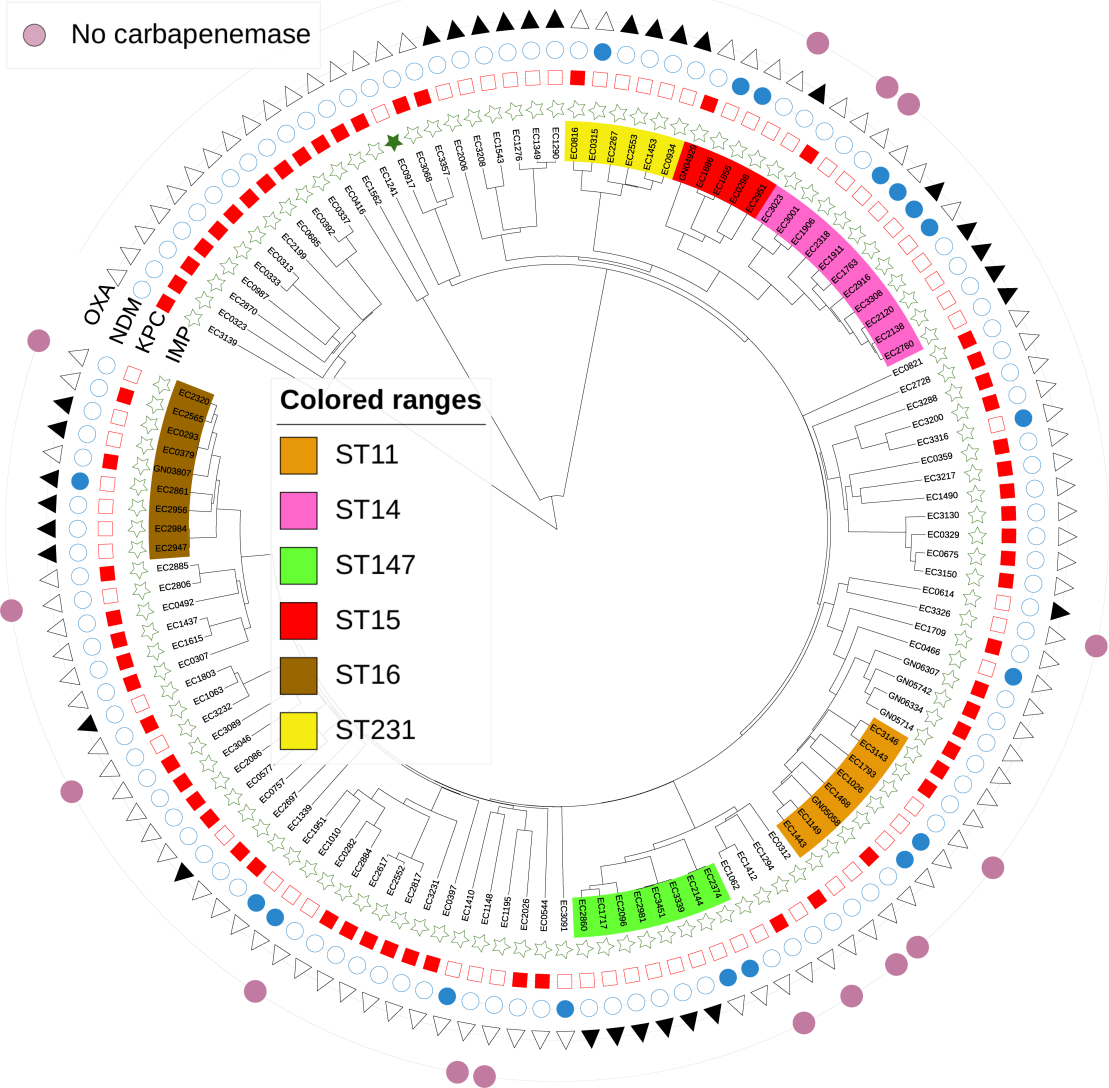
Phylogenetic relationships and distribution of carbapenemases in CRKp isolates in this study. STs with five or more CRKp isolates are indicated as internal colored ranges. The presence of IMP (filled green star), KPC (filled red square), NDM (filled blue circle), and OXA (filled black triangle) carbapenemases is indicated through filled symbols. The lack of these enzymes is indicated through open symbols. CRKp isolates without a carbapenemase are highlighted with a purple circle in the outer ring.

Of the 125 CRKp isolates used in this study, 109 (87%) contained a carbapenemase. A total of 107 CRKp isolates had a single carbapenemase; 61 (57%) contained a KPC (55 KPC-2 and 6 KPC-3), 29 (28%) contained an OXA (16 OXA-181, 9 OXA-232, and 4 OXA-48), 16 (16%) contained an NDM (13 NDM-1, 2 NDM-4, and 1 NDM-7), and 1 (1%) contained IMP-1. Two isolates contained two carbapenemases; one strain contained OXA-232 and NDM-1, and the other contained OXA-181 and NDM-5. Figure 2 details the phylogenetic relationship between the CRKP isolates in the study population, as well as the distribution of carbapenemases within the population.

The majority of carbapenemase-producing CRKp isolates encoded a concomitant extended-spectrum beta-lactamase-type (ESBL) enzyme (82/109 [75%]). Of these 82 isolates, 18 had more than one type of ESBL. CTX genes were identified in 70 CRKp isolates, and these were almost entirely CTX-M-15 (*n* = 65). ESBL-type SHV genes were identified in 22 CRKp isolates, with SHV-9 (*n* = 17) occurring most commonly.

### Mobilizability of carbapenemases

To determine the potential transmissibility of carbapenemases between bacterial strains, we characterized the local genomic context of each carbapenemase (i.e., plasmid vs chromosomal) and, if plasmid encoded, the potential for plasmid transfer to another strain (i.e., conjugative, mobilizable, or nonmobilizable). The local genomic context could be determined for 107 (96%) of the carbapenemases. Of these, 96% (103/107) were plasmid encoded. The four chromosomal carbapenemases were all OXA-type enzymes (three OXA-181 and one OXA-232). Among the plasmid-encoded carbapenemases, the majority were conjugative (82/103 [80%]) or mobilizable (11/103 [11%]). The carbapenemases encoded within the remaining 10 non-mobilizable plasmids were not particular to any one enzyme class (three KPC-2, three NDM-1, three OXA-181, and one NDM-5).

### Non-carbapenemase-producing CRKp

Of the 125 CRKp isolates included in this study, 16 (13%) contained no detectable carbapenemase by genetic or phenotypic examinations. In these 16 isolates, all but one had at least one type of ESBL. CTX genes were identified in 15 isolates, with CTX-M-15 (*n* = 8) and CTX-M-3 (*n* = 4) most common. SHV-9 genes were found in two isolates.

Prior work has shown that in the absence of carbapenemase enzymes, the combination of ESBL enzymes and loss of porins *ompK35* and/or *ompK36* can drive resistance to carbapenems (27–29). Therefore, we examined the presence of porin mutations in this cohort of CRKp isolates. Of the 16 isolates with no detectable carbapenemase, mutations in the porin genes *ompK35* and *ompK36* were identified in 14 (88%). Truncated versions of both *ompK35* and *ompK36* mutations were reported in four (25%) isolates, a truncated version of *ompK35* alone was identified in one (6%) isolate, and truncated versions of *ompK36* alone were identified in nine (56%) isolates. In carbapenemase-containing CRKp isolates, mutations in *ompK35* and/or *ompK36* were less often present (42/109 [39%] vs 14/16 [88%]; *P* = 0.0003). RT-PCR analyses also revealed varying degrees of decreased transcript levels of *ompK35* and/or *ompK36* in all carbapenemase-negative isolates, suggesting that porin loss/deficiency may have a role in mediating carbapenem resistance in these isolates (Fig. S1).

### Comparison of CRKp infections in patients with and without a carbapenemase

Characteristics of patients with CRKp infections from isolates that did and did not contain a carbapenemase are shown in Table 3. Patients with CRKp infections from isolates containing a carbapenemase were older than those that lacked a carbapenemase (carbapenemase: mean 64 years [SD 16]; non-carbapenemase: 55 [17]; *P* < 0.0001). Patients with CRKp infections from isolates containing a carbapenemase, relative to those that lacked a carbapenemase, had increased total in-hospital mortality (carbapenemase: mortality 54/109 [50%]; non-carbapenemase: mortality 3/16 [19%]; *P* = 0.03) and attributable mortality (carbapenemase: mortality 32/109 [29%]; non-carbapenemase: mortality 1/16 [6%]; *P* = 0.04). The increased mortality in the carbapenemase group did not appear to be driven by one type of enzyme (i.e., KPC, NDM, etc.). Given the association between carbapenemase production and increased mortality, we generated multivariable logistic regression models of total mortality and attributable mortality. Using these multivariable models, we found that the presence of a carbapenemase was associated with both increased total mortality (odds ratio 5.2; 95% confidence interval 1.5–25.5; *P* = 0.02) and attributable mortality (odds ratio 9.2; 95% confidence interval 1.6–176.2; *P* = 0.04; Table S2).

**TABLE 3:** Characteristics of patients with carbapenemase-producing *K. pneumoniae* vs non-carbapenemase-producing *K. pneumoniae^a^*

Characteristics	Carbapenemase *n* (%) *N* = 109	No carbapenemase *n* (%) *N* = 16	*P*-value
Age in years (mean [SD])	64 (16)	55 (17)	**<0.0001**
Female	39 (36)	5 (45)	0.79
Infection source			0.59
Blood	66 (61)	11 (69)	
Lower respiratory	43 (39)	5 (31)	
Patient outcomes			
Total mortality	54 (50)	3 (19)	**0.03**
Attributable mortality	35 (32)	1 (6)	**0.04**
Septic shock	31 (28)	3 (19)	0.55
Capsule type			0.36
KL15	10 (9)	2 (13)	
KL64	12 (11)	0	
KL2	6 (4)	2 (13)	
K51	6 (6)	1 (6)	
KL10	5 (5)	0	
Other	70 (64)	11 (69)	
Virulence score			0.11
0	49 (45)	10 (63)	
1	34 (31)	6 (38)	
2	4 (4)	0	
3	6 (6)	0	
4	9 (8)	0	
5	7 (6)	0	

^*a*^ *P*-values less than 0.05 are indicated in bold.

### Additional antibiotic resistance genetic elements

Along with the carbapenemase and ESBL genes described above, the CRKp isolates in this study contained a high number of additional antibiotic resistance genes and antibiotic resistance-associated mutations. The CRKp isolates contained genes conferring resistance to a median of nine antibiotic classes (interquartile range 6–10). Resistance to these antibiotic classes was conferred through the presence of a median number of 11 antibiotic resistance genes (IQR 8–14). The distribution of resistance to antibiotic classes and antibiotic resistance genes is shown in Fig. S2A and B, respectively. A schematic of antibiotic resistance genes contained in each CRKp isolate is shown in Fig. S3. Genes conferring reduced susceptibility to commonly used antibiotic classes were common: fluoroquinolones (110/125; 88%), aminoglycosides (104/125; 83%), trimethoprim (93/125; 74%), and sulfonamides (93/125; 74%). Genes conferring resistance to colistin (9/125; 7%) or fosfomycin (2/125; 2%) were rare.

### Capsule types and virulence genes

Of the 125 isolates in this study, capsule types could be determined by analysis of *wzi* alleles in 111 cases. There was significant diversity, with 44 capsule types identified. Just six capsule types were present in at least five CRKp strains: KL15 (*n* = 12), KL64 (*n* = 12), KL2 (*n* = 8), KL51 (*n* = 7), and KL10 (*n* = 5). The capsule type was highly correlated with ST by a *χ*^2^ test (*P* < 0.0001), though not associated with the presence of a carbapenemase gene (*P* = 0.36). We did not identify any significant associations between capsule type and patient outcomes of septic shock, attributable mortality, or total mortality.

The CRKp isolates also varied widely in virulence gene content. A virulence gene score could be calculated based on the number of virulence genes in each CRKp isolate. Virulence scores may range from a minimum value of 0 to a maximum value of 5. The majority of CRKp isolates in this study had a virulence score of 0 (*n* = 59) or 1 (*n* = 40), though scores from 2 to 5 were also observed (Fig. S4). The presence of individual virulence factors also revealed variability: aerobactin (*n* = 21), colibactin (*n* = 9), salmochelin (*n* = 11), yersiniabactin (*n* = 60), *rmpABC* (*n* = 9), and *rmpA2* (*n* = 14). Neither virulence score nor the presence of any individual virulence gene was associated with the patient outcomes of septic shock, attributable mortality, total mortality, or culture site (i.e., lower respiratory tract vs bloodstream infection). Interestingly, there was a trend toward increasing odds of containing a carbapenemase gene with increasing virulence score, though this finding did not meet statistical significance (Odds ratio [OR] 1.98; 95% confidence interval [CI] 0.96–4.09; *P* = 0.06). Incorporation of the virulence score into the previously generated multivariable regression models of total and attributable mortality (Table S2) revealed continued associations between the presence of carbapenemase and increased mortality and no such associations between virulence score and mortality.

### Analysis of recurrent CRKp isolates

**Fig 3:**
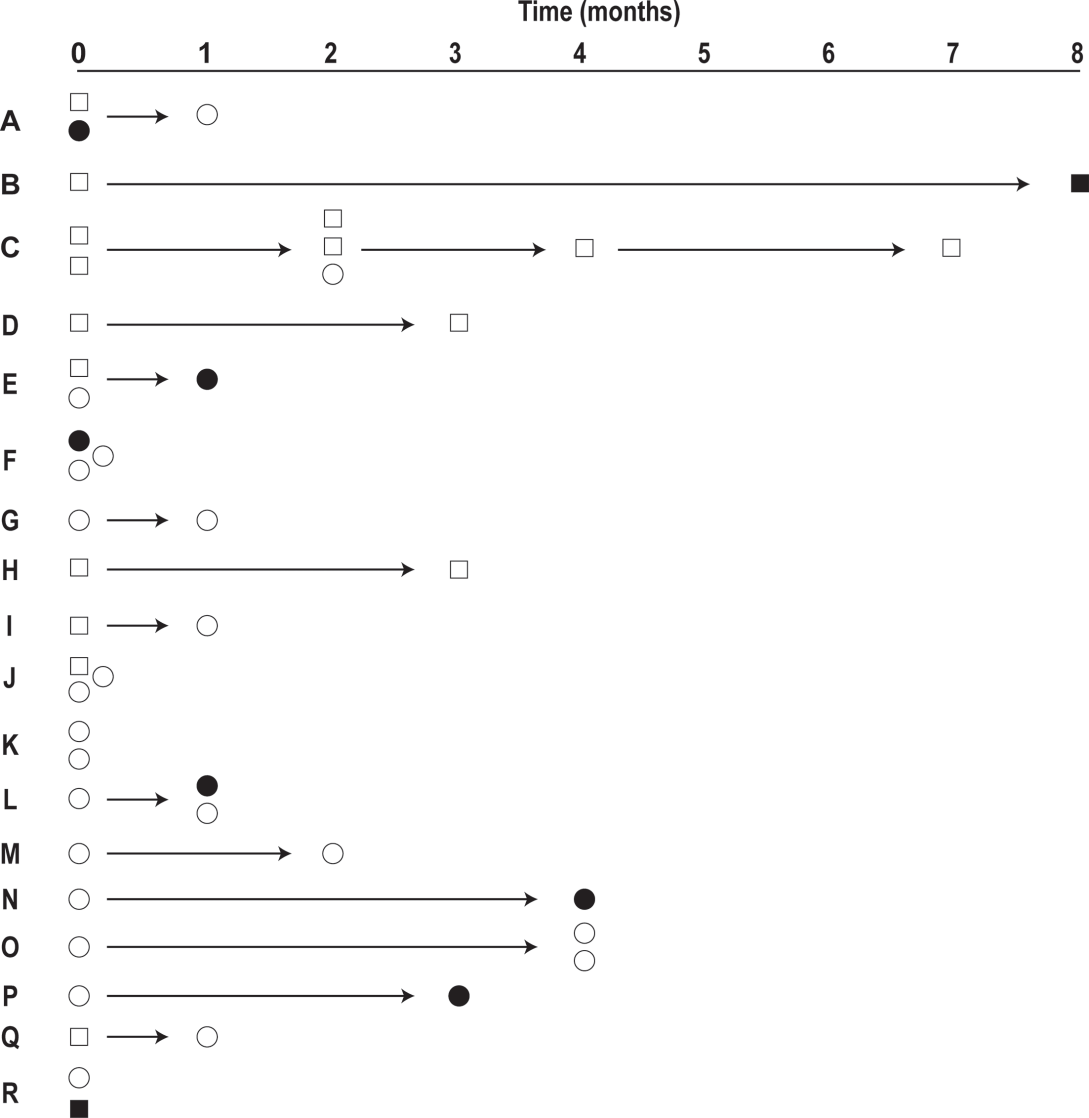
Genetic analysis of recurrent CRKp infections. Eighteen patients (A through R) in this study had multiple instances of CRKp isolation from the blood (circles) or respiratory tract (squares). The time (months) from the initial CRKp isolation to the subsequent CRKp-positive cultures is indicated by the x-axis. CRKp isolates that are the same genomic group as the initial infection are shown in white, and CRKp strains that differ from the initial are shown in black.

The analyses above focused on single CRKp isolates from the blood or respiratory tract of enrolled patients. However, 18 (15%) patients had multiple CRKp isolates cultured from the blood or respiratory tract over the study period. There were 47 CRKp isolates (18 initial; 29 follow-up) from these 18 patients. Genetic analysis of the recurrent CRKp isolates revealed that the majority were the same genomic group as the initial isolate (20/29 [69%]; Fig. 3). In the most extreme case, a single patient had seven CRKp isolates of the same genomic group that were cultured from blood and the respiratory tract over an 8-month period. Interestingly, in cases of recurrent CRKp infections with different strains (*n* = 9), the different CRKp strains isolated from the same patient commonly contained a similar plasmid. In these nine cases of recurrent CRKp with a different strain, we could identify the genomic context of the carbapenemases in nine cases. In most of these cases (5/6 [83%]), the recurrent CRKp strain contained a similar carbapenemase-encoding plasmid as the CRKp causing the initial infection. Thus, there is consistency in the plasmid content of CRKp causing recurrent infections, even if the initial and second infections are caused by different strains.

The 20 recurrent CRKp isolates of the same genomic strain as the initial isolate were isolated from 12 patients as some patients had multiple recurrences with the same genomic group. There were no clear microbiological factors driving these recurrences. The 12 recurrences involved CRKp isolates from 12 STs (i.e., each had a unique ST) and 10 capsule types. The 12 recurrences consisted of strains both with (*n* = 10) and without (*n* = 2) a carbapenemase. Though the KPC-2 gene was common in the recurrent cases (8/12 [75%]), this did not meet statistical significance given the small sample size (recurrent with the same strain: 8/12 contained KPC-2 [75%]; all others: 47/113 contained KPC-2 [42%]; *P* = 0.13).

## DISCUSSION

In this study, we aimed to understand how carbapenem resistance emerges in a large and diverse set of CRKp isolates from the blood and respiratory tract of patients in Singapore and the United States. We further aimed to understand how the mechanism of carbapenem resistance, as well as other clinical and bacterial characteristics, impacts patient outcomes in this cohort. The genomic epidemiology of CRKp in Singapore has been previously described in detail in several publications (12, 13). These prior studies included a large proportion of non-infection-causing colonizing isolates, and CRKp isolates driving invasive infections have been less well understood. Here, we focused on CRKp-causing blood and respiratory tract infections as they are associated with poor clinical outcomes. In total, we identified significant diversity in mechanisms of carbapenem resistance and in the underlying STs in these clinically relevant blood and respiratory CRKp isolates. We further determined that the mechanism of carbapenem resistance was associated with patient mortality in this cohort. Each of these is discussed in detail below.

First, we observed considerable diversity in the mechanisms of carbapenem resistance. At the most basic level, resistance to carbapenems was identified in *K. pneumoniae* strains both with and without a carbapenemase. In strains with a carbapenemase, we observed enzymes from four major classes (KPC, OXA, NDM, and IMP) and nine sub-classes (e.g., KPC-2, KPC-3, etc.), with the most common sub-class being present in only ~50% of such isolates. This diversity in mechanism is similar to prior studies comprising CRKp from all isolation sites from Singapore (12, 13), though different from other regions that have a single dominant enzyme. For example, studies from the United States and China have demonstrated a preponderance of KPC-type enzymes (84%–100%) in carbapenemase-producing CRKp strains (14, 30–32). In strains without a carbapenemase, the primary mechanism of carbapenem resistance appeared to be a combination of ESBL enzymes with mutations in the porins *ompK35/ompK36*. This combination has been previously demonstrated to influence susceptibility to carbapenems (27–29). The porins *ompK35* and *ompK36* have been demonstrated to be important in the diffusion of antibiotics into the bacterial cell (33), and the loss of one or more of these porins presumably decreases the intracellular drug concentration. Interestingly, the mechanisms of carbapenem resistance demonstrated significant diversity even within individual CRKp STs. For example, the most common ST—ST14 (*n* = 10)—generated resistance to carbapenems through KPC-2, NDM-1, OXA-232, and through no detectable carbapenemase.

Second, we observed significant clonal diversity among the CRKp isolates. Remarkably, the three most common STs together only accounted for 22% of the CRKp isolates in the study. Almost two-thirds of the identified STs were represented by just a single CRKp isolate. Again, significant clonal diversity was previously noted in CRKp isolates from Singapore (12), whereas other studies have demonstrated dominant clones such as ST11 in China (14, 30) and ST258 in the United States (14, 34). The high underlying genetic diversity of the CRKp isolates in this cohort may reflect Singapore’s role as an international hub for business, medicine, and tourism. Carbapenemase-resistant bacteria have been demonstrated to be present in environmental sources such as water reservoirs and meat in Singapore (35, 36).

Third, we found that patients with carbapenemase-producing CRKp infections had significantly higher total mortality and attributable mortality than patients with non-carbapenemase-producing CRKp infections. This is despite the fact that there were relatively few patients with non-carbapenemase-producing CRKp infections (*n* = 16). The impact of carbapenemase presence on mortality is an area of controversy. In patients with carbapenem-resistant Enterobacterales infections, the presence of a carbapenemase, relative to lack of carbapenemase, has been associated with increased mortality in one study (37), decreased mortality in another study (38), and no difference in mortality in other studies (39–41). Ultimately, it is challenging to compare such differences in outcomes across multiple cohorts for several reasons. The prior studies primarily involved patients with bacteremia (37–40) and/or addressed infections due to multiple different carbapenem-resistant species of Enterobacterales (37–39, 41). In our cohort, the increased mortality in patients with carbapenemase-containing CRKp was driven in large part by patients with pneumonia, a population not addressed in prior studies. In addition, geographic variation in CRKp molecular epidemiology and both the timing and nature of appropriate antibiotic therapy likely confound our ability to understand well the impact of the mechanism of carbapenem resistance on patient outcomes.

This study had several limitations. First, the majority of the CRKp isolates (>90%) came from a single center in Singapore. However, the overall findings of high clonal diversity and variation in mechanisms of carbapenem resistance are consistent with prior studies from this region. Second, the overall low number of patients limited our ability to generate firm conclusions regarding the molecular epidemiology of CRKp and associations between particular genetic elements and clinical outcomes. Third, information regarding antibiotic therapy was not incorporated into our models of patient mortality, and this may be an important confounding factor that could account for the observed differences in clinical outcomes among patients with CRKp infections due to strains with and without a carbapenemase. Finally, we focused on the bloodstream and respiratory tract isolates due to the high mortality associated with these infections, though we did not address the molecular epidemiology of CRKp causing colonization or less invasive infections.

In conclusion, our study illustrates the complexity of managing patients with CRKp bloodstream infections and/or pneumonia. As we and many others have shown, these infections are associated with high mortality. The high mortality is due in large part to the challenge of appropriate antibiotic therapy. In many cases of CRKp infection, appropriate antibiotic therapy depends in large part on the mechanism of carbapenem resistance. For example, newer antibiotics such as ceftazidime-avibactam or meropenem-vaborbactam may be appropriate therapy for patients with KPC-producing organisms, though not for NDM-producing organisms given the stability of the enzyme in the presence of these drugs. Patients with CRKp infection due to an NDM-producing organism may benefit from combination therapy with antibiotics such as aztreonam and ceftazidime-avibactam (42), though the success of such a regimen requires detailed knowledge of the particular beta-lactamases that are present. There is no clear optimal antibiotic therapy for patients with infections due to non-carbapenemase-containing CRKp infections. Ultimately, the complexity of carbapenem resistance, as illustrated in this study, speaks to the importance of rapid diagnostics that provide not only antibiotic susceptibility patterns but also the presence of clinically relevant beta-lactamases and porin mutations. Such information would likely improve how quickly appropriate antibiotic therapy can be delivered and possibly improve patient outcomes.

## Data Availability

The samples are published under Bioproject PRJNA715288. The biospecimens span from SAMN18344174 to SAMN18344327.

## SUPPLEMENTAL MATERIAL

Supplemental material spectrum.00631-25-s0001.docx Supplemental methods, Tables S1 and S2, and Fig. S1 to S4.

ASM does not own the copyrights to Supplemental Material that may be linked to, or accessed through, an article. The authors have granted ASM a non-exclusive, world-wide license to publish the Supplemental Material files. Please contact the corresponding author directly for reuse.
