# Supplementary material for: Molecular epidemiology and clinical characteristics of carbapenem-resistant Klebsiella pneumoniae bloodstream and pneumonia isolates
Source: Microbiol Spectr. 2025 Jul 9;13(8):e00631-25. doi: 10.1128/spectrum.00631-25 (PMC12323367; doi:10.1128/spectrum.00631-25)
Supplement: Supplemental material — Supplemental methods, Tables S1 and S2, and Fig. S1 to S4. [file spectrum.00631-25-s0001.docx]

**Supplemental Methods.**

**Quantitative reverse transcription polymerase chain reaction (qRT-PCR).** All PCR amplification reactions were set up with 5μL Bio-rad Universal iTaq Universal SYBR Green Supermix kit (Bio-Rad, USA), 1 microliter cDNA (0.5 ng), 0.5μM forward and reverse primers, and appropriate nuclease-free water to a total volume of 10 microliters per reaction. The thermal cycler conditions were as follows: One initial polymerase activation and DNA denaturation cycle for 30s at 95°C, and 40 amplification cycles (denaturation for 2s at 95°C, annealing/extension for 15s at 63°C). Melting curve analyses were also performed for each run.

**Supplemental Table 1. Primers used in this study for the qRT-PCR experiments.**

| **Gene** | **Primer** | **Sequence (5’→ 3’)** |
| --- | --- | --- |
| *rpoB* | rpoB-F | GCCGCGACCAGGTTGA |
| *rpoB* | rpoB-R | GCACGGTTGGCGTCATC |
| *ompK35* | ompK35-F | AACGGCAACAAACTGGACTTCT |
| *ompK35* | ompK35-R | GGCCGATACGGGCATAGG |
| *ompK36* | ompK36-F | CGACCAGACCTACATGCGTGTA |
| *ompK36* | ompK36-R | GTTGTTCGCCTGAACGTTGTATT |

**Supplemental Table 2**. Multivariable logistic regression models of variables associated with total mortality and attributable mortality (i.e., death due to infection as opposed to other causes) in patients with carbapenem-resistant *Klebsiella pneumoniae* bloodstream or lower respiratory tract infections.

|  | **Total mortality** | | | **Attributable mortality** | | |
| --- | --- | --- | --- | --- | --- | --- |
| **Variable** | **OR** | **95% CI** | **P-value** | **OR** | **95% CI** | **P-value** |
| Age | 0.99 | 0.97-1.02 | 0.57 | 0.99 | 0.96-1.02 | 0.44 |
| Female sex^1^ | 0.58 | 0.25-1.29 | 0.19 | 0.49 | 0.19-1.20 | 0.13 |
| Lower respiratory source^2^ | 1.74 | 0.79-3.90 | 0.17 | 1.19 | 0.50-2.82 | 0.69 |
| United States^3^ | 0.52 | 0.07-2.66 | 0.36 | 0.39 | 0.02-2.59 | 0.40 |
| Carbapenemase-containing^4^ | 5.24 | 1.45-25.53 | **0.02** | 9.24 | 1.63-176.21 | **0.04** |
| Virulence score | 0.93 | 0.72-1.19 | 0.57 | 0.94 | 0.71-1.22 | 0.67 |
| ^1^ Reference is male sex  ^2^ Reference is isolates from the blood  ^3^ Reference is patients in Singapore  ^4^ Reference is isolates without a carbapenemase gene | | | | | | |

**Supplemental Figure 1.** Expression of *ompK35* and *ompK36* in non-carbapenemase-producing CRKp isolates (n=16) relative to a reference strain (*K. pneumoniae* ATCC 13883). Expression was determined by RT-PCR. The log2-transformed fold change of gene expression in the clinical isolates relative to the reference strain is shown. No *ompK35* expression in one CRKp isolate or *ompK36* expression in another CRKp isolate could be detected, so this data is not shown here as log2 of 0 is undefined.

**
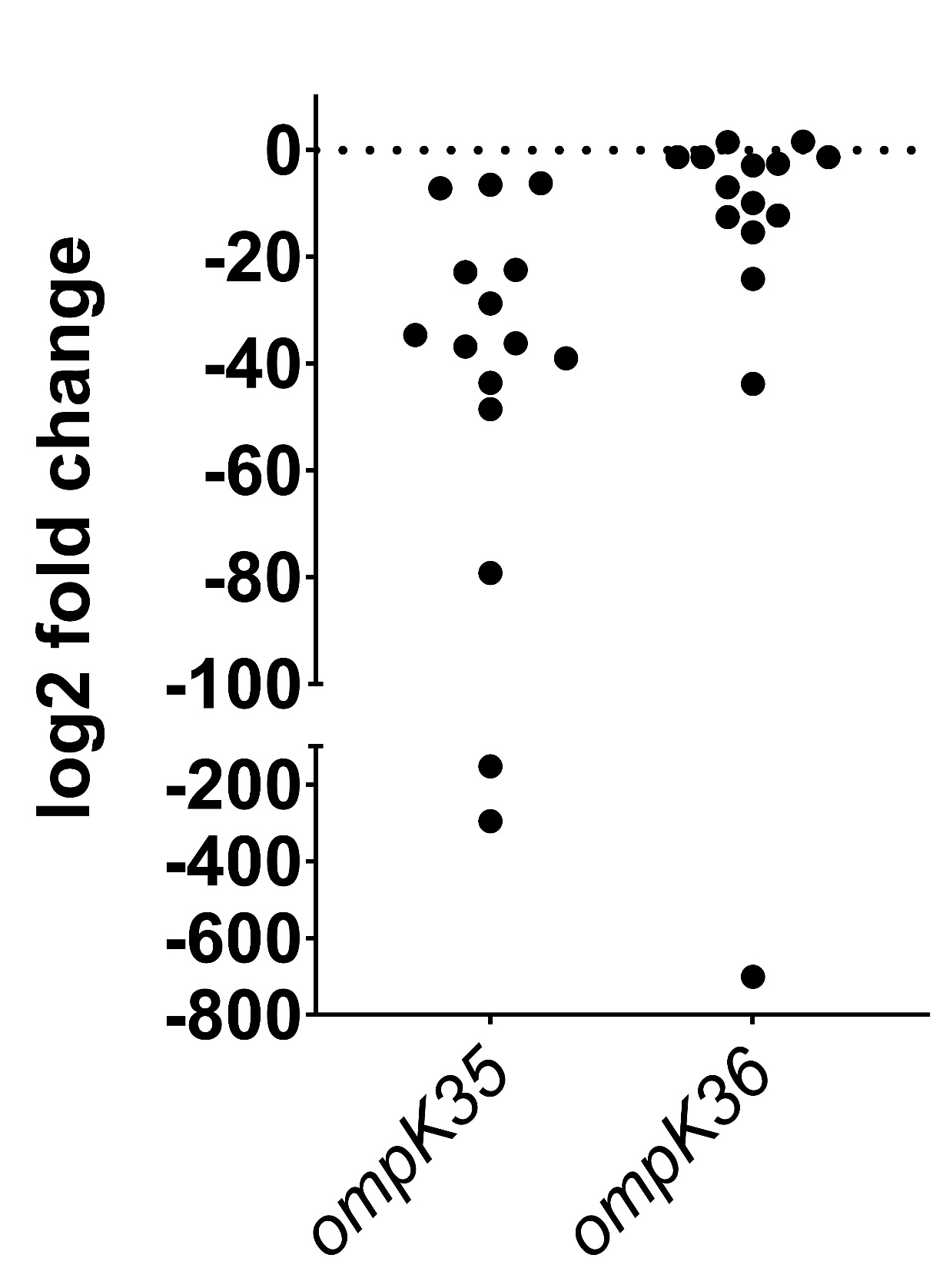
**

**Supplemental Figure 2.** Each carbapenem resistant *Klebsiella pneumoniae* (CRKp) isolate is resistant to a particular number or antibiotic classes and contains a particular number of antibiotic resistance genes. Shown here are the distributions of the resistance to numbers of antibiotic classes (A) and the numbers of antibiotic resistance genes (B).


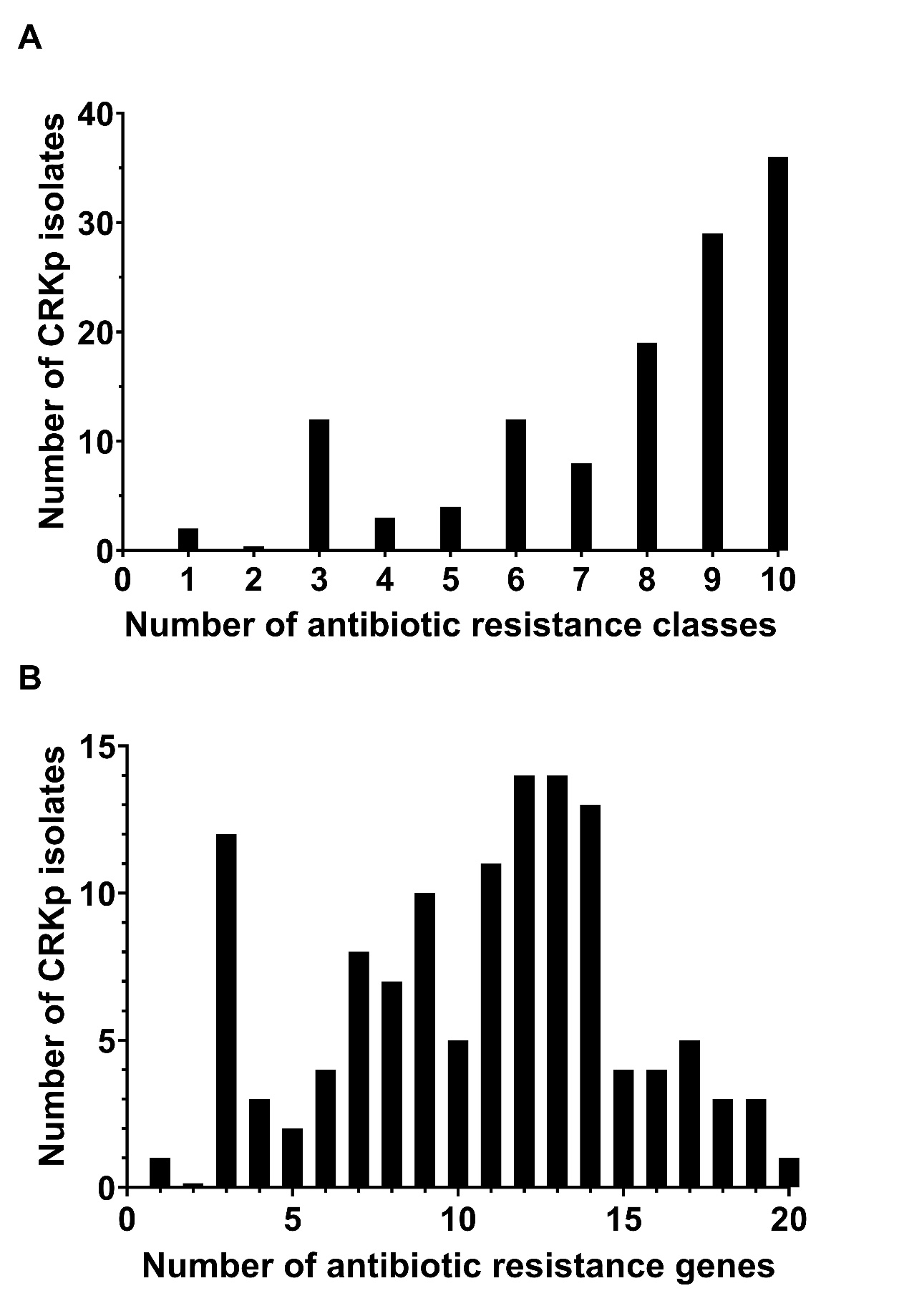


**Supplemental Figure 3. Antibiotic resistance genes (non beta-lactamase) in carbapenem-resistant *Klebsiella pneumoniae* bloodstream infection and pneumonia isolates.** The six most common sequence types (ST11, ST14, ST147, ST15, ST16, ST231) are indicated with internal colored ranges. The presence of genes contributing to resistance to aminoglycosides (red), rifampin (dark blue), chloramphenicol (green), erythromycin (yellow), fosfomycin (pink), fluoroquinolones (black), trimethoprim (light blue), sulfonamide (orange), and tetracycline (purple) are indicated.

**
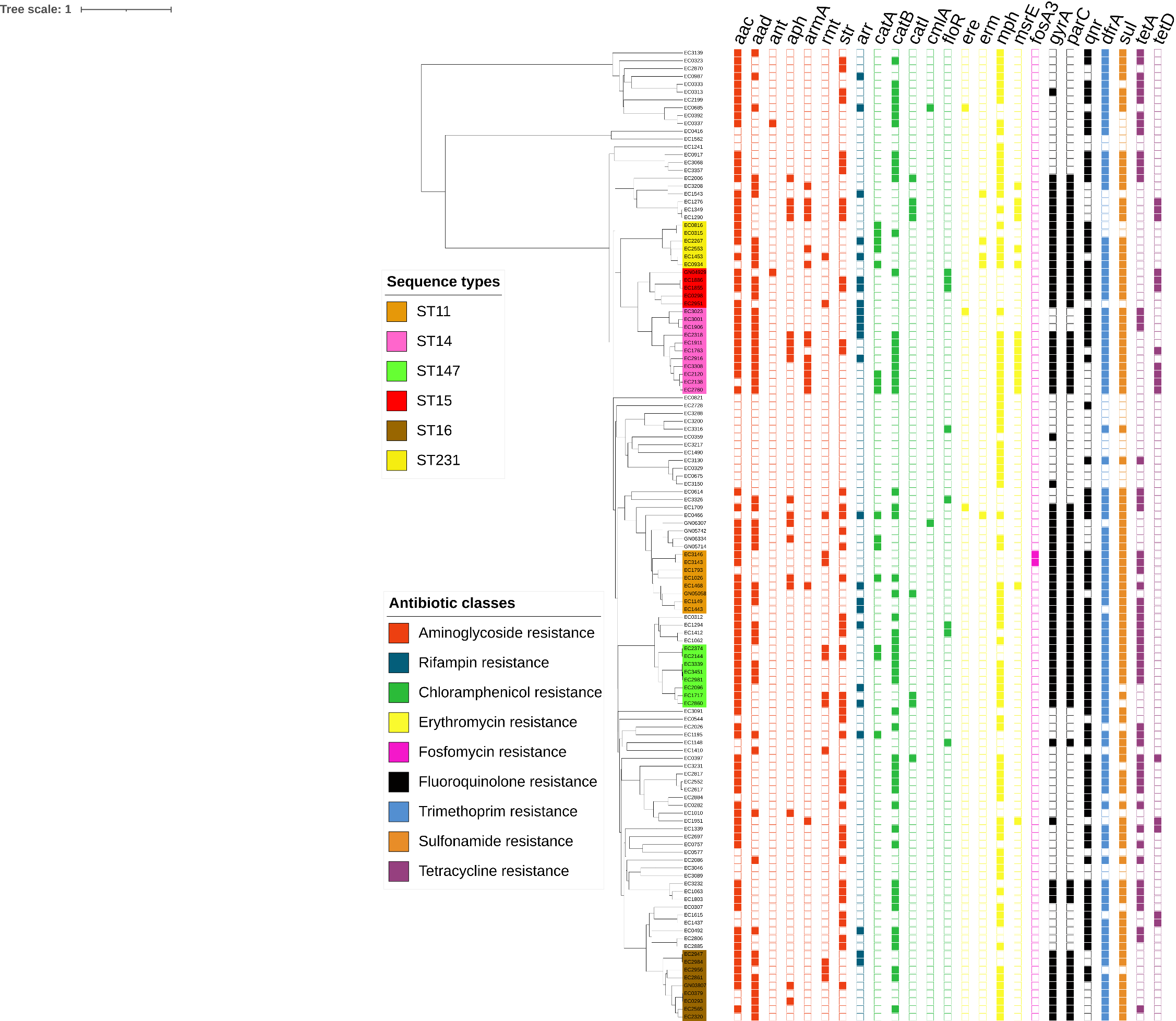
**

**Supplemental Figure 4. Distribution of virulence scores among carbapenem-resistant *Klebsiella pneumoniae* (CRKp) isolates.** Virulence score ranges from 0 to 5 is determined by the presence of key virulence genes [1].


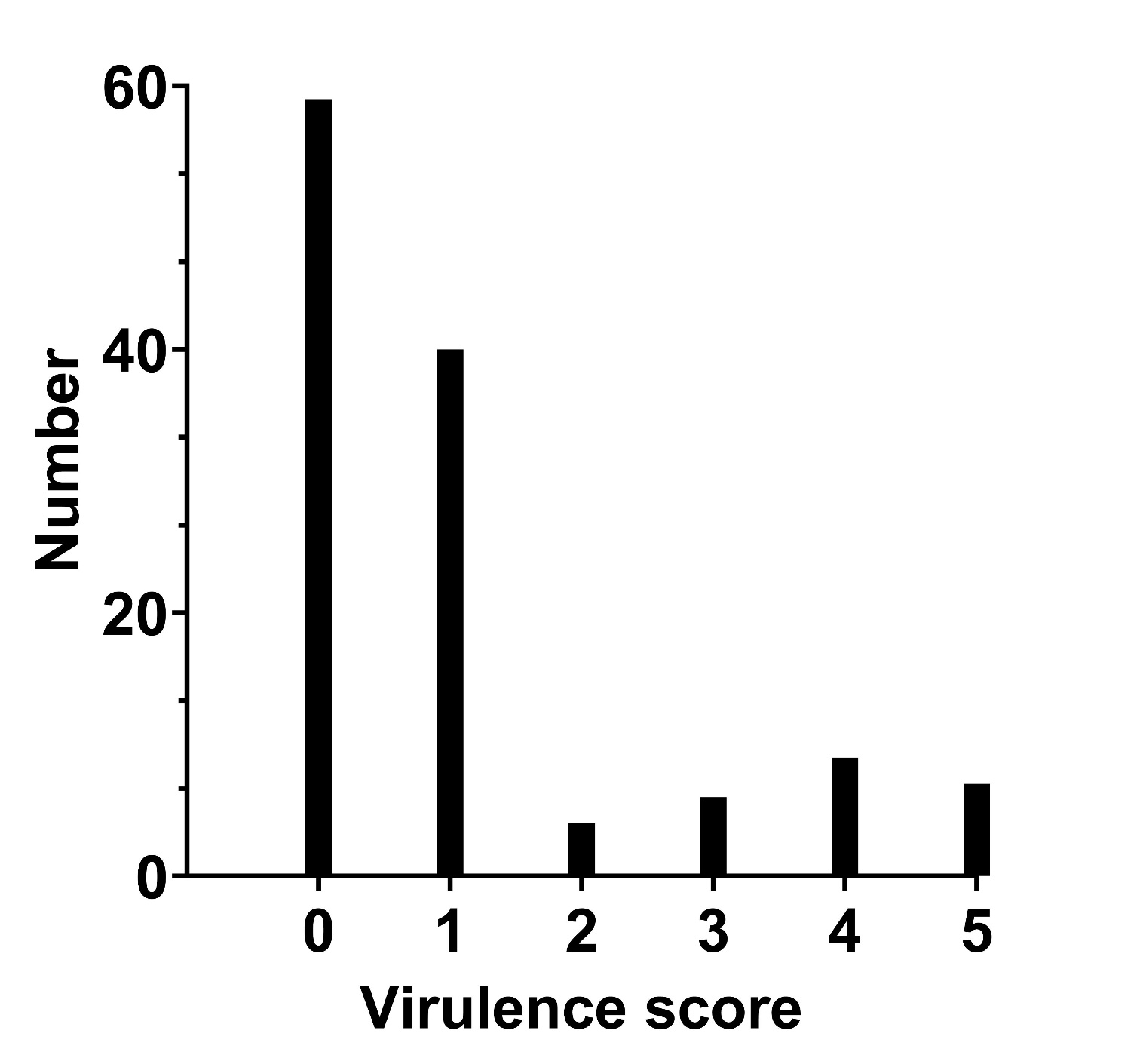


**References**

1. Lam, M.M.C., et al., *A genomic surveillance framework and genotyping tool for Klebsiella pneumoniae and its related species complex.* Nat Commun, 2021. **12**(1): p. 4188.
